# Quantum Metrology: Surpassing the shot-noise limit with Dzyaloshinskii-Moriya interaction

**DOI:** 10.1038/srep16360

**Published:** 2015-11-09

**Authors:** Fatih Ozaydin, Azmi Ali Altintas

**Affiliations:** 1 Department of Information Technologies, Isik University, Istanbul, Turkey; 2 Department of Electrical Engineering, Okan University, Istanbul, Turkey

## Abstract

Entanglement is at the heart of quantum technologies such as quantum information and quantum metrology. Providing larger quantum Fisher information (QFI), entangled systems can be better resources than separable systems in quantum metrology. However the effects on the entanglement dynamics such as decoherence usually decrease the QFI considerably. On the other hand, Dzyaloshinskii-Moriya (DM) interaction has been shown to excite entanglement. Since an increase in entanglement does not imply an increase in QFI, and also there are cases where QFI decreases as entanglement increases, it is interesting to study the influence of DM interaction on quantum metrology. In this work, we study the QFI of thermal entanglement of two-qubit and three-qubit Heisenberg models with respect to SU(2) rotations. We show that even at high temperatures, DM interaction excites QFI of both ferromagnetic and antiferromagnetic models. We also show that QFI of the ferromagnetic model of two qubits can surpass the shot-noise limit of the separable states, while QFI of the antiferromagnetic model in consideration can only approach to the shot-noise limit. Our results open new insights in quantum metrology with Heisenberg models.

The influences of spin-orbit coupling on non-classical correlations play an important role in foundations of quantum mechanics and quantum information. Zhang showed that arising from the spin-orbit coupling, the Dzyaloshinskii-Moriya (DM) interaction^1^,^2^,^3^ can excite the thermal entanglement of Heisenberg model both in the ferromagnetic and antiferromagnetic cases and also that quantum teleportation is realized better with the ferromagnetic model, than the antiferromagnetic model, if DM interaction exists^4^. Various models of two and three level systems, some including homogeneous and even inhomogeneous external magnetic fields have been examined in detail, revealing how the DM interaction overwhelms the decoherence effects on entanglement due to thermalization and external magnetic fields^5^,^6^,^7^,^8^,^9^.

A key field that entangled systems can outperform separable systems is metrology. That is, the quantum Fisher information (QFI) of a separable system of *N* particles can be at most *N*, achieving the so-called shot-noise limit (SNL). On the other hand, QFI of an entangled system of *N* particles *can* exceed SNL (and therefore the system is so called *useful* for surpassing SNL in quantum metrology), scaling as *N*^2^ in the ideal case, which is the fundamental or so-called Heisenberg limit (HL)^10^, achievable by pure GHZ states. Pezzé and Smerzi proposed a condition for particle entanglement, i.e. for an *N* qubit state, if *χ*^2^, the ratio of *N* to the QFI of the state is smaller than 1, then the state is multiparticle entangled. However, pure multipartite entanglement alone is not sufficient for achieving HL and even surpassing SNL^11^. What is more, decoherence due to inevitable interactions with the environment decreases the QFI of an open system in general. This gave rise to devoting an intense effort on QFI with the motivation of finding which systems are *useful* under which conditions^12^,^13^,^14^,^15^,^16^,^17^,^18^,^19^,^20^,^21^,^22^. Recently it was shown that GHZ-like bound entangled states can be *useful*^23^ and the behavior of QFI and the geometric discord of a three-level bound entangled system are similar^24^.

Although *χ*^2^ (or its reciprocal, *QFI per particle*) can detect multiparticle entanglement, it cannot be a direct entanglement measure since it is not monotonic under local operations and classical communications (LOCC) but there have been efforts on finding a relation between QFI and entanglement measures^25^, as well as on finding entanglement measures based on QFI^26^.

An increase in the entanglement of a state does not imply an increase in the QFI of the state. Consider a general GHZ state of *N* qubits under decoherence, for example. As the strength of decoherence decreases from maximum to zero, the entanglement of the state increases. However, Ma *et al.* showed that as the strength of the decoherence decreases from maximum to a critical point, *i*) under the amplitude damping, QFI does not increase but rather decreases, and *ii*) under phase damping, QFI stays constant at 1, the SNL of QFI per particle^17^.

Therefore a natural question arises: Does the existence of DM interaction excites QFI as it excites entanglement? A more interesting question is whether DM interaction can help a *non-useful* state to become *useful* for sub-shot-noise sensitivity in quantum metrology. In this work, we study the quantum Fisher information of thermal entanglement of two-qubit and three-qubit Heisenberg models. The Hamiltonian of the models with DM interaction we consider is


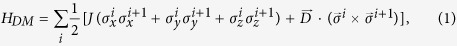


where 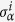 is the Pauli matrix 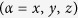 applied to 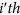 particle, *J* is the coupling constant and 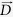 is the vector coupling, which we choose in *z* direction, for simplicity.

We show that DM interaction excites QFI in general, making the Heisenberg models *better resources* in quantum metrology even at high temperatures. We also show that in the two-qubit case in consideration, if DM interaction exists, a *non-useful* Heisenberg model in the ferromagnetic region can become *useful* (surpassing the shot-noise level), while in the antiferromagnetic region, the model can only approach to the shot-noise level.

## Results

In two-qubit Heisenberg model, the eigenvalues and the associated eigenvectors of the Hamiltonian *H*_*DM*_ are found as 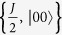, 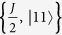, 
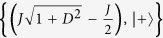
, and 
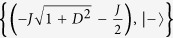
, where 
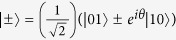
 and 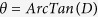. It is easy to see that introducing the DM interaction excites the entanglement of the system. Taking the Boltzmann constant *k* = 1 and 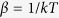, the density matrix of the Heisenberg model in the thermal equilibrium as a function of temperature (T) can be found by 
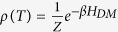
, where 
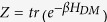
 is the partition function and 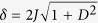. The non-zero elements of 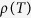 are found as


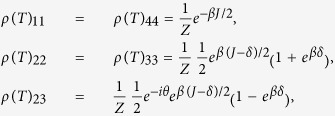


and


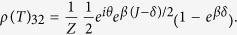


The eigenvalues and the associated eigenvectors, 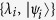 of 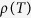 are


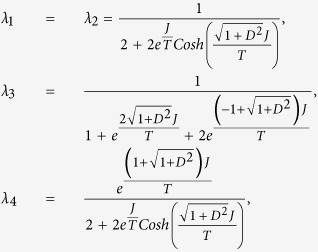


and


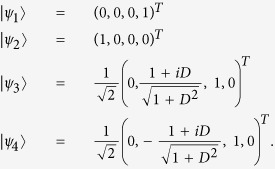


Using the eigenvalues and eigenvectors of 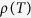, we obtain the quantum Fisher information of the system per particle, 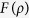 via Eq. (4) with respect to temperature *T*, the spin coupling coefficient *J* and the strength of DM interaction *D* as


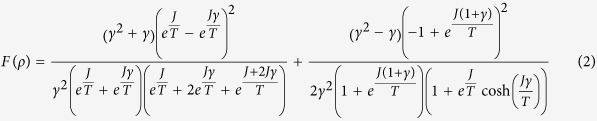


where 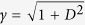.

**Figure 1:**
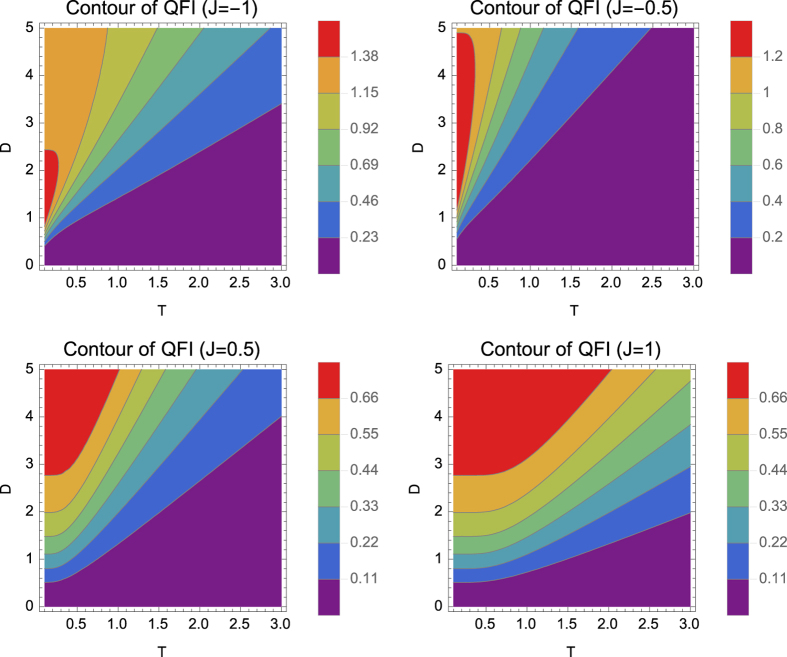
Quantum Fisher information (QFI) per particle (in the units of Boltzmann constant *k*) for ferromagnetic (*J* < 0) and antiferromagnetic (*J* > 0) two-qubit Heisenberg models with respect to temperature (*T*) and strength of DM interaction (*D*) (in the units of Boltzmann constant *k*). In both cases, DM interaction increases the QFI of the models, surpassing the shot-noise limit of separable states in the ferromagnetic model only.

**Figure 2:**
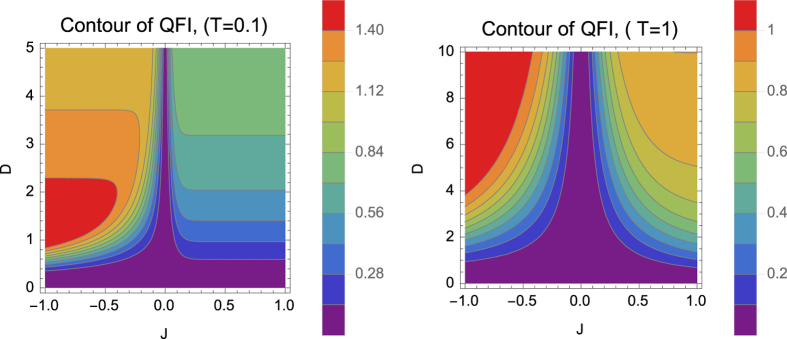
Quantum Fisher information (QFI) per particle for *T* = 0.1 (left) and *T* = 1 (right) with respect to *D* and *J*. DM interaction excites the QFI of the models, surpassing the shot-noise limit of separable states in the ferromagnetic model only. However the increase of DM strength does not imply a continuous increase the QFI, as shown in the low temperature case.

In Fig. 1, we show that for the fixed values of *J*, if DM is not introduced, even at low temperatures, QFI of the Heisenberg models vanish. However, as introduced with an increasing strength, DM interaction overwhelms the effect of thermalization, resulting a considerable increase in the QFI for both ferromagnetic and antiferromagnetic models. It is surprizing that in the ferromagnetic range, the model becomes and stays *useful* as *D* increases, i.e. 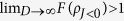. However this is not the case for antiferromagnetic model. Even at very low temperatures, increase of the strength of DM interaction cannot make the system *useful*, i.e. 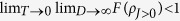. In Fig. 2, we show that for two fixed values of temperature, i.e. 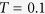 and 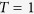, in the presence DM interaction, QFI of both models are exited but the model becomes useful only in the ferromagnetic range.

**Figure 3:**
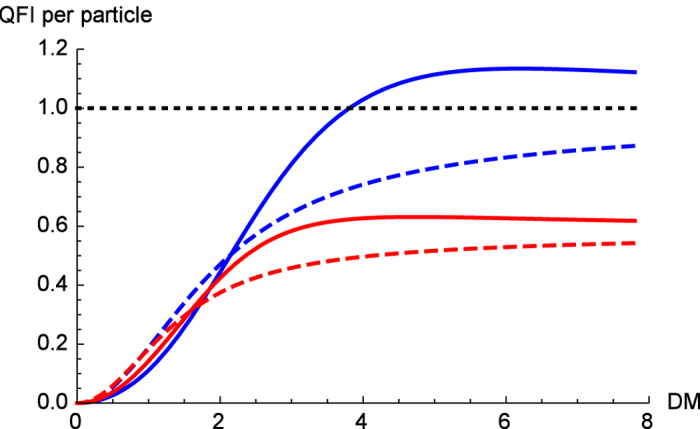
Quantum Fisher information per particle for the Heisenberg model of two (blue) and three (red) qubits in the ferromagnetic (solid) and antiferromagnetic (dashed) regions. Dotted line is for the shot-noise level of the separable states.

When it comes to three-qubits, although the DM interaction excites the QFI, the Heisenberg model fails to become *useful* in both ferromagnetic and antiferromagnetic cases, no matter how low the temperature or how high the DM interaction is. Due to the lengthy terms of the Hamiltonian and the corresponding density matrix, we skip the intermediate steps and present our numerical results in Fig. 3.

## Discussion

We have shown that the Dzyaloshinskii-Moriya interaction excites the quantum Fisher information of the two-qubit and three-qubit Heisenberg models, overwhelming the thermalization effects both in the ferromagnetic and in antiferromagnetic regions.

We have also shown that for the two-qubit case, in the ferromagnetic region, as the interaction strength increases, the system becomes *useful* for surpassing the shot-noise level of the separable states in quantum metrology. This result is surprizingly in accordance with the result of Zhang i.e. quantum teleportation is realized better with ferromagnetic chain than antiferromagnetic chain, in the presence of DM interaction^4^. The two-qubit model we have chosen in the first place is the simplest spin chain, yet powerful such that it can be used for constructing a quantum computer^28^. We have found that as the number of qubits forming the thermal entanglement of the Heisenberg model increases beyond two, the sensitivity of the system per particle in quantum metrology gradually decreases. This is not surprizing since it was shown by Hyllus *et al.*^11^ that even for the pure states, as the number of particles *N*, forming a one or even two dimensional cluster state increases, in particular for *N* > 4 the QFI per particle of the model can only achieve the shot-noise level. Therefore one would not expect a sub-shot-noise sensitivity in quantum metrology with the Heisenberg models of more than four qubits, even enhanced by the Dzyaloshinskii-Moriya interaction. Therefore we have limited the number of particles of the Heisenberg model by less than four. It would be interesting to study the effects of external magnetics fields and DM interaction on more complex chains for quantum metrology. We believe that our work open new insights in quantum metrology with magnetic spin chains.

## Methods

In quantum metrology, a common scenario is that the parameter *ϕ* to be estimated is introduced to an initial state *ρ* by a transformation, obtaining *ρ*(*ϕ*) to be measured. Then the parameter *ϕ* is estimated from the measurement results. In the literature^11^,^27^ the transformation is usually associated with the Mach-Zenhder interferometer, where the transformation is a phase transformation achieved by the collective spin operators 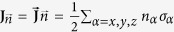 in the direction 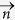 with 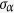 the Pauli operators.

The variance of the estimation of the parameter *ϕ* of a density matrix *ρ*(*ϕ*) is limited by the Cramer-Rao bound^27^.


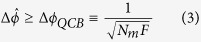


where 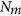 is the number of experiments, *F* is the quantum Fisher information and the estimator 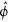 satisfying 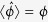. Considering that the parameter *ϕ* is obtained by a SU(2) operation, i.e. 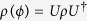 with 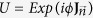. Therefore 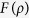, the maximal quantum Fisher information per qubit over the directions of a state *ρ* can be found by^17^


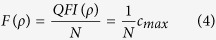


where *N* is the number of qubits of the state and *c*_*max*_ is the largest eigenvalue of the symmetric matrix *C* of which elements are given as


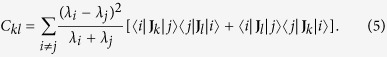


Here, 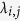 and 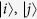 are the eigenvalues and the associated eigenvectors of the density matrix of the state and 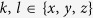. Note that the above definition of maximal QFI per particle is a sufficient condition for entanglement i.e. 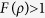, as introduced by Pezze and Smerzi^10^ in the reciprocal form, i.e. 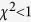 and the above method of calculating the maximal QFI per particle of a state is used for exploring the conditions where the state becomes useful for sub-SNL metrology. Ma *et al.* calculated maximal QFI per particle of GHZ states under three basic decoherence channels and also the critical points where the maximal QFI per particle of GHZ states surpass SNL, i.e. 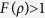, with respect to the strength of decoherence^17^.

For the Heisenberg model in consideration, we find 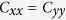 and 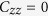. Therefore maximal QFI of the *N*-qubit model per qubit can be found as 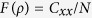. As 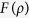 exceeds SNL (i.e. 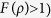 the state is regarded as *useful* for quantum metrology.

## Additional Information

**How to cite this article**: Ozaydin, F. and Altintas, A. A. Quantum Metrology: Surpassing the shot-noise limit with Dzyaloshinskii-Moriya interaction. *Sci. Rep.* **5**, 16360; doi: 10.1038/srep16360 (2015).
